# Epiphyseal Sparing and Reconstruction by Frozen Bone Autograft after Malignant Bone Tumor Resection in Children

**DOI:** 10.1155/2015/892141

**Published:** 2015-12-21

**Authors:** Ahmed Hamed Kassem Abdelaal, Norio Yamamoto, Katsuhiro Hayashi, Akihiko Takeuchi, Shinji Miwa, Hiroyuki Tsuchiya

**Affiliations:** ^1^Department of Orthopedic Surgery, Graduate School of Medical Science, Kanazawa University, Kanazawa 920-8641, Japan; ^2^Department of Orthopedic Surgery, Faculty of Medicine, Sohag University, Sohag 82524, Egypt

## Abstract

Limb salvage surgery has become the standard treatment for malignant primary bone tumors in the extremities. Limb salvage represents a challenge in skeletally immature patients. Several treatment options are available for limb reconstruction after tumor resection in children. We report our results using the technique of epiphyseal sparing and reconstruction with frozen autograft bone in 18 children. The mean follow-up period for the all patients included in this study is 72 ± 26 m. Eight patients remained disease-free, seven patients lived with no evidence of disease, two were alive but with disease, and one patient died of the disease. Five- and ten-year rates of survival were 94.4%. Graft survival at 5 and 10 years was 94.4%. Functional outcome using the Enneking scale was excellent in 17 patients (94.4%) and poor in one patient (5.5%). Complications include 2 nonunions, 2 fractures, 2 deep infections, 1 soft tissue recurrence, and leg length discrepancy in 7 cases. This technique is a good reconstructive choice in a child with a nonosteolytic primary or secondary bone tumor, responsive to chemotherapy, without involvement of the articular cartilage. It is a straight forward, effective, and biological technique, which affords immediate mobilization of joints and possible cryoimmune effects, with excellent long term functional outcome and less complication.

## 1. Introduction

Limb salvage has changed from being an exception to standard practice in the management of primary malignant bone tumors [1]. The majority of patients can be cured using a multidisciplinary approach which includes a treatment team of oncologists, radiation oncologists, surgeons, pathologists, and radiologists and enrollment of patients in clinical trials [2]. Limb salvage surgery represents a challenge in skeletally immature patients in whom further growth is anticipated [3]. The selected treatment method should address the current bone defect and the expected leg length discrepancy (LLD) at maturity. Surgeons have several choices for the reconstruction of large bone defects after tumor resection, for example, endoprostheses, allografts, vascularized fibular grafts, composite arthroplasty, distraction osteogenesis, or biological reconstruction [4]. Biological reconstruction by reusing the resected tumor bearing bone is steadily increasing, through the use of extracorporeal irradiation [1], autoclaving [5], pasteurization [6], or freezing [7]. The common advantage of these techniques is the coincidence of configuration of the bone defects and the reconstructive material, so that the reconstructive procedure can be performed relatively easily [8]. Yamamoto et al. described the use of freezing to treat the bone containing the tumor using liquid nitrogen at −196°C, which was used as a cryogenic agent used to destroy the tumor cells [7]. Freezing devitalizes tumor cells by inducing ice crystal formation and cell dehydration. Only one cycle of −196°C for 20 minutes is sufficient to kill all tumor cells [7]. A second cause of cell death during cryosurgery is ischemic infarction due to thrombosis of the microcirculation [9].

Epiphyseal sparing tumor resection surgery has been attempted in recent years. This is likely attributable to better imaging technologies and more experience with limb preservation techniques. The advantages of this technique are preservation of a normal joint in a young patient, the avoidance of joint complications seen with osteoarticular grafts (need for conversion to TKA at some point, joint instability), and no need for endoprostheses (loosening, revisions) [10].

In our study we evaluated the long term results of epiphyseal preservation and reconstruction by frozen tumor bearing autograft bone in 18 patients with malignant bone tumors in childhood.

## 2. Patients and Methods

Since 1999 the musculoskeletal tumor division of our orthopedic department has performed greater than 150 cases of biological reconstruction using freezing bone technique, including 36 cases in children using different reconstruction techniques, that is, osteoarticular frozen autograft, composite frozen bone tumor prosthesis, and intercalary freezing. In this study, we reviewed the long term results of epiphyseal preservation and reconstruction by intercalary frozen autograft bone.

*Level of Evidence*. Level of evidence was level IV therapeutic study.

*Study Design*. This was a retrospective clinical review study.

The inclusion criteria of our study were patients 18 years old or younger, primary nonosteolytic malignant bone tumors, with no extension to the epiphysis on MRI, effective preoperative chemotherapy, length of the planned bone recycling that is only limited by the possibility to achieve rigid fixation of the frozen bone to the host bone, and rigid stabilization using locked or nonlocked plates, or intramedullary nails. The mean age was 12 ± 3.4 y (6–18 y) with nine children younger than 12 years and nine adolescents (12–18 years); they were nine girls and nine boys.

The mean follow-up period for all patients included in this study was 72.8 ± 26.5 m (32–155 m). The pathological diagnosis was osteosarcoma in 16 patients, Ewing's sarcoma in 1 patient, and undifferentiated round cell sarcoma in 1 patient. The tumor lesion was in the femur in nine cases, in the tibia in eight cases, and in the calcaneus in one case. All patients received preoperative and postoperative chemotherapy [11, 12]. The study had ethical approval from the institutional review board of the Kanazawa University and a written informed consent was obtained from the child guardians on behalf of their child in every case of this study.

Demographic criteria of all patients included in this study are listed in Table 1.

### 2.1. The Surgical Procedure

Tumor resection was carried out by en bloc resection. Wide resection was performed in nine cases and marginal resection in nine cases, and subchondral level of osteotomy was based on the tumor extension on MRI. A minimum of twenty millimeters of tumor free subchondral bone is necessary for epiphyseal sparing reconstruction and ten millimeters of subchondral bone is needed to allow screw fixation. A ten-millimeter or greater resection beyond the tumor extension on MRI is optimal. Intraoperatively, isolation of the tumor bearing bone is carried out using surgical sheets, and removal of soft tissue component and curettage of medullary cavity are then performed. The tumor bearing bone is frozen using liquid nitrogen for 20 minutes, thawed at room temperature for 15 minutes, and lastly rinsed with 37°C distilled water for 15 minutes.

**Figure 1:**
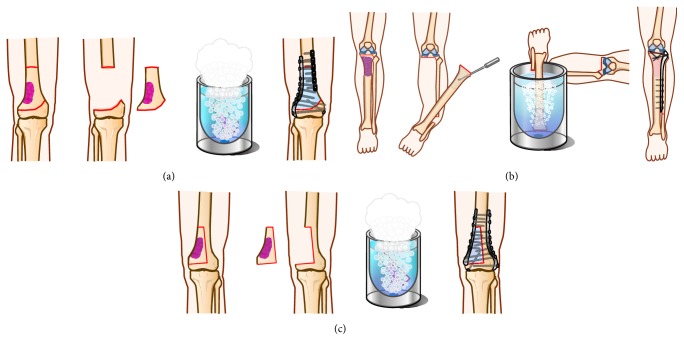
Illustration showing different methods and technique of freezing. (a) Free freezing (intercalary) in distal femur. There are two osteotomies and the tumor bearing bone is totally immersed in liquid nitrogen with no anatomical continuity with the host bone. (b) Pedicle freezing (intercalary) in the tibia. Also this figure shows the joint preservation technique through performing the osteotomy in subchondral bone. (c) Free freezing (hemicortical) in the tibia. The osteotomy line surrounds the lesion and the tumor bearing bone is freely frozen, while there is an anatomical continuity in the host bone proximal and distal to the tumor.

Different kinds of freezing techniques are used according to the number and sites of osteotomies. “Free freezing” is a term used when two osteotomies are done and the tumor bearing bone is totally immersed in liquid nitrogen with no anatomical continuity between the diseased and healthy host bone (Figure 1(a)). Free freezing was carried out in 11 cases.

The term “pedicle freezing” is used when only one osteotomy is performed and the freezing by immersion in liquid nitrogen is carried out with anatomical continuity of the tumor bearing bone with the host bone at one of its two ends (Figure 1(b)). This technique is suitable whenever the tumor location is proximal tibia or proximal femur [13]; it was carried out in seven cases. “Hemicortical freezing” was carried out in three cases when the tumor extension on MRI allowed performing the osteotomy around the lesion. The tumor bearing bone is freely frozen, while maintaining anatomical continuity of the host bone proximal and distal to the tumor (Figure 1(c)). Fixation of the osteotomies was performed after freezing. Intramedullary nailing was carried out in three cases and platting in 14 cases. Fixation was performed using one plate in three cases, two plates in nine cases, and three plates in two cases. Fixation using only lag screws was performed in one case. Soft tissue reconstruction was performed and the patellar tendon reattached. The wound was closed over suction drains.

Patients were allowed range of movement (ROM) exercises immediately postoperatively. Touch-down weight bearing using crutches was allowed two months after surgery and weight bearing protection was continued until sufficient callus at the junction between normal and frozen bone is seen radiographically. Full weight bearing was allowed when solid union was evident.

Functional evaluation of the patients was performed using the revised 30-point functional classification system established by the International Society of Limb Salvage and the Musculoskeletal Tumor Society (MSTS). The functional score measured six parameters: pain, function, emotional acceptance, use of walking supports, walking ability, and gait. Each parameter is given a value ranging from 0 to 5, according to specific criteria. The individual scores are added together to obtain an overall functional score, with a maximum of 30 points, which then is expressed as a percentage of normal, with 30 points being defined as normal function. A score of 23 points or greater is considered an excellent functional result, 15 to 22 points a good result, 8 to 14 points a fair result, and less than 8 points a poor result [14].

### 2.2. Statistical Analysis

Autografts that were functional and viable were considered as having “survived,” and those that were removed were considered as having “died.” Survival of autografts was recorded using the Kaplan-Meier method with 95% confidence interval. The follow-up period is calculated from the date of surgery until the last follow-up visit; no patient was lost to follow-up.

## 3. Results

**Figure 2:**
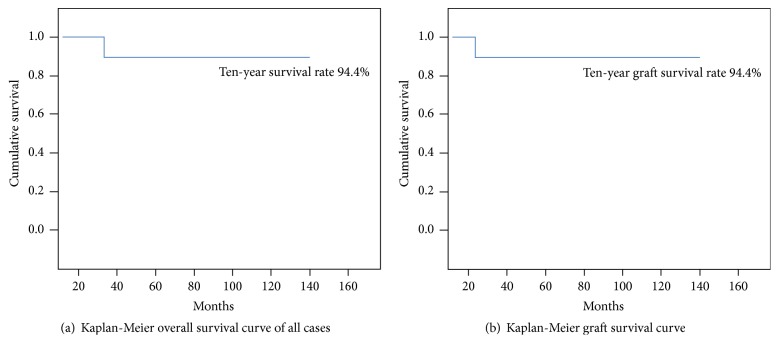
Survival curves. (a) Kaplan-Meier survival curve showing the five- and ten-year overall survival. (b) Kaplan-Meier survival curve showing the graft five and ten-year survival.

A total of eight patients remained disease-free at last follow-up, seven patients lived with no evidence of disease, two patients were alive but with disease, and one patient died of their disease. Five- and ten-year rates of survival were 94.4%. Five- and ten-year survival rates of the graft were 94.4% (Figure 2).

Function on the MSTS score was excellent in 17 patients (94.4%) and poor in one (5.5%). Union was achieved in 16 out of the 18 cases (88.8%) with the average time to union being 8.6 ± 2.5 months (6–15). There was no statistical significance difference between the mean union times in cases who had pedicle freezing and those who had free freezing, 8.5 and 8.7 months, respectively (*p* > 0.05). The three cases of hemicortical resection all united, with a mean union time of 7.3 ± 0.4 months. Hemicortical resection provided an excellent option with good results. The lowest complication rate was seen in this group with no recurrences, fractures, infections, or nonunions. This group also had the shortest mean time to union. There were two cases that had nonunion, both from the free freezing group. Nonunion was encountered in two cases and they were treated by allograft augmentation at the nonunion site.

Local recurrence from the soft tissue part occurred in one case (5.5%), it was complicated by deep infection and finally treated by above knee amputation, and no recurrence had occurred within the frozen bone or in the preserved epiphyseal bone. Lung metastasis was evident in the first visit in two patients (11.1%), while four patients developed lung metastases later on (22.2%); thoracoscopic excision and/or open thoracic excision of the metastases or the lung segment(s) was carried out by thoracic surgeons. Fracture of the graft occurred in two cases (11.1%); they were managed by osteosynthesis and both fractures eventually united. Deep infection occurred in two cases (11.1%); in one case we performed medial gastrocnemius muscle flap after surgical debridement, and in the other case infection was accompanied by the former listed recurrence.

**Figure 3:**
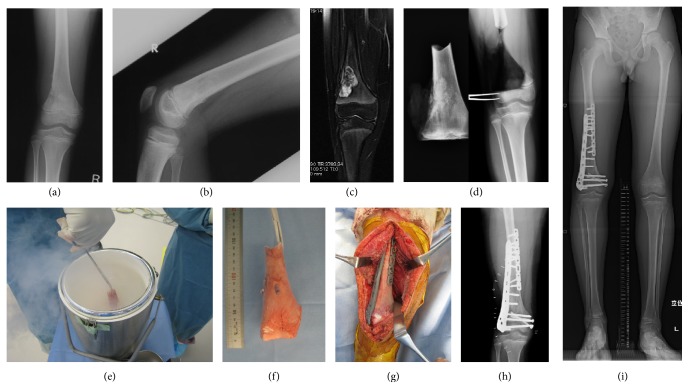
Case presentation representing a ten-year-old boy at time of surgery with osteosarcoma distal right femur. (a, b) Anteroposterior and lateral view of distal femur XR showing the tumor mass. (c) T2 weighed MRI image of the distal femur showing the tumor mass with high signal intensity. (d) Intraoperative XR showing the freely resected segment to be frozen and the host bone. (e) Intraoperative photo of free freezing. (f) The tumor bearing bone after freezing. (g) The frozen segment after repositioning and fixation. (h) Postoperative XR of the distal femur after freezing and fixation. (i) Long length film showing a LLD of 3 cm.

Leg length discrepancy (LLD) was encountered in seven patients with average LLD at last follow-up being 22 mm (7–32 mm). The LLD was corrected in four cases by limb lengthening using Taylor spatial frame (TSF) if the LLD > 20 mm. If it was less than 20 mm, as in the other three cases, they were managed conservatively by shoe lifts and carefully watched for changes in LLD and for functional adaptation. Two cases had varus deformity in addition to LLD and were corrected simultaneously by TSF. A representative case is presented in Figure 3; a thirteen-year-old boy with osteosarcoma of his distal femur had undergone a free frozen autograft, which fully united. He is 3 years postoperative at the time of writing this paper and has a LLD of 3 cm and he is scheduled for limb lengthening surgery using TSF.

## 4. Discussion

Cryosurgery was first used in the management of bone tumors at the Memorial Sloan-Kettering Cancer Center in the United States in 1964 as a palliative procedure on a patient with a metastasis to the humerus from primary lung cancer [16, 17]. Marcove et al. [18] reported the use of liquid nitrogen for the treatment of osteosarcoma in 1984. They used repetitive freezing and thawing to destroy tumor cells present at the margin of the curettage. They reported no evidence of residual tumor though en bloc excision of the tumor was not performed at that time. Many authors have described the use of cryosurgery for the management of benign and malignant bone tumors [19].

Yamamoto et al. [7] documented the efficacy of treatment with liquid nitrogen on osteosarcoma cells, in vitro and in vivo; additionally, they found that frozen autografts maintained adequate biomechanical properties. Takata et al. [22] reported that bone morphogenetic activity was better preserved in frozen autografts treated by liquid nitrogen than in those treated with autoclaving or pasteurisation.

Among the different surgical modalities which are available for limb reconstruction in children, the use of intraoperative freezing by liquid nitrogen has multiple advantages; it is a simple technique, has lower cost, preserves osteoinduction and osteoconduction [22], exhibits no graft rejection, does not transmit disease, does not degrade biomechanical strength [7], allows easy attachment of tendons and ligaments to bone, does not contain harmful denatured substances, and provides early revitalization with possible cryoimmunological effects. The procedure requires less equipment and requirements compared to heat or radiation treated bone grafts [23].

Overall survival rate in our cases was 94.4% at five and ten years. This survival rate is higher compared with other studies. Aponte-Tinao et al. [10] reported survival rate of 86% in a study that included 35 patients treated by epiphyseal preservation and allograft reconstruction; Campanacci et al. [21] reported a survival rate of 72.2% at five years in a study including 19 children treated by allograft-prosthetic composite for proximal tibial reconstruction. Picardo et al. [3] reported a survival rate of 80% in their series of 55 children treated by noninvasive extendible endoprosthesis.  Table 2 summarizes the demographic data and results of different reconstruction methods used in children.

Our relatively high survival rate may be partially attributed to the strict selection criteria of this procedure, performing this procedure in only good responders to neoadjuvant chemotherapy.

Local recurrence rate in our series is comparable to other methods of reconstruction; Hong et al. [1] reported markedly low local recurrence rates after limb preservation surgery with extracorporeal irradiation in a large series of 101 patients. They reported local recurrence rates of 2.9%, 0%, and 20% for patients with Ewing sarcoma, osteosarcoma, and chondrosarcoma, respectively. They however reported relatively higher distant recurrence rates of 22.9%, 16.6%, and 30% for the same tumors. Picardo et al. [3] reported no local recurrences in their series. It is noteworthy that no recurrence occurred in cases that had marginal resection, while the only case of recurrence had occurred in the group of cases that had a wide marginal resection. This is consistent with Jeon et al. [24] who reported 35 local recurrences in 445 osteosarcomas and could not find relationship between adequacy of soft tissue margin and local recurrence in the corresponding area.

Fracture of the graft in our series was less than other techniques. Aponte-Tinao et al. [10] reported fracture of the allograft in 11 of 35 patients (31%). Campanacci et al. [21] reported six fractures in nineteen allografts (32%). Yu et al. [25] reported three fractures in five patients (66%) treated with preservation of the epiphysis after resection of high-grade osteosarcomas and reconstruction using inactivated bone.

Infection is one of the main challenges in tumor surgery. The infection rate in our series was comparable to or less than other reconstruction techniques. Picardo et al. [3] reported infections in 10.9% of their cases, using noninvasive lengthening techniques. The infection rate was even higher in a study using an extendible endoprosthesis (47%) [20]. The infection rate was less in other reports of reconstruction using allografts with and without epiphyseal preservation (5.7% and 5%) [10, 21].

Although there was no significant difference in the union time between pedicle and free frozen cases, the occurrence of nonunions was greater in the free frozen group compare to the pedicle frozen group (22.2% versus 0%); Shimozaki et al. [26] previously reported a lower complication rate in the pedicle frozen as compared to free frozen cases in a comparative study. In our series, overall complication rate was 33.3%. However, our small sample size means that even one or two complications might have resulted in a much higher percentage of complications.

Particular attention should be paid to LLD as a late sequel in pediatric tumor surgery; it is an inevitable outcome when the physis is affected by the tumor or surgical resection. The level of activity and functional outcome is largely dependent upon how the LLD is managed in addition to other factors [20]. Lengthening is performed when the LLD is >20 mm and is performed in the “virgin” bone; that is, if the frozen bone is the femur, we lengthen the tibia, and vice versa. This is because full revitalization of the frozen bone takes up to six years [27], and the goal is to attain a biomechanically stable regenerate. Additionally, we do not prefer to interfere with the fixation procedure and we prefer to avoid masking of any local recurrence. Lengthening was performed by distraction osteogenesis using the Taylor spatial frame (TSF), and distraction is started at the standard time point after the osteotomy; the distraction process was uneventful.

Hemicortical resection was first described in 1982 by Campanacci et al. [28] for surgical management of patients who had parosteal osteosarcoma of the distal femur and in 2012 by Chen et al. [29] who reported the long term outcome of hemicortical resection and biological reconstruction in the treatment of high grade sarcoma in six patients. Hemicortical resection provided excellent results in our cases. In our three cases who underwent hemicortical resection, we observed the least complication rate, and additionally the patients had the shortest mean time to union. Hemicortical resection necessitates accurate preoperative evaluation of the three-dimensional extension of the tumor using CT and MRI to be able to accurately plan the osteotomy lines.

Functional outcomes in this series are encouraging with excellent results achieved in 94.4% of cases comparable to the results of minimally invasive and noninvasive extendible prosthesis, intercalary resection and allograft reconstruction, and resurfaced allograft prosthesis composite reconstruction [3, 10, 20, 21].

Different reconstructive alternatives should be available to the surgeon and young patient, and allografts may represent a good reconstructive option if availability is not an issue. The evolution of noninvasive expandable prostheses alleviates the high rate of complication because lengthening is painless and can be done without anesthesia in an outpatient clinic. Unfortunately, this lengthening system involves the use of a magnet in the implant, and thus MRI cannot be used to scan the patient for possible tumor recurrence. In addition to the common complications of arthroplasty, continued lengthening may be limited in an effort to avoid the risk of fixed flexion deformity and development of neurapraxia. Results of extracorporeal irradiation are also promising; however, no reconstruction procedure is without drawbacks. Availability is an issue at many centers, and surgeon has to adapt the reconstruction procedure for each case according to the tumor extension, response to chemotherapy, and patient choice.

Tumor response to preoperative neoadjuvant chemotherapy is an important prognostic facto, especially if the patient is to enroll into the recent EURAMOS protocol where postoperative chemotherapy is dependent on the tumor response to neoadjuvant chemotherapy [30]. The drawbacks of this technique include the inability to perform histological analysis of the entire specimen; however, partial histopathological examination is always done to assess the response to neoadjuvant chemotherapy according to the Rosen and Huvos grading system, [15] (Table 1). Miwa et al. reported that although tumor cellularity and the degree of necrosis due to chemotherapy are heterogeneous throughout the tumor, the presumed disadvantage of this technique is the histological assessment of the small and prereconstruction sample from the graft may not be representative of the cellularity and necrosis of the tumor as a whole. They analyzed the correlation between histological response and prognosis in osteosarcoma patients who underwent reconstructive surgery using frozen tumor bearing autografts. They found that the histological response determined from the graft biopsy was adequate and representative of the whole tumor and correlated with the overall survival [31].

Though Yamamoto et al. [7] reported there is no significant difference in compression strength between the intact bone and the bone treated with the one-cycle liquid nitrogen process, they determined that liquid nitrogen-treated bone has sufficient initial strength for limb reconstruction, comparable to that of allografts and pasteurized bones; however, the presence of a lytic lesion too often weakens the resected specimen and it lacks the eligibility for using this technique. If the osteolytic lesion is small enough and lies in non-weight bearing bone, (i.e., humerus), it might be possible to perform this technique, with adequate internal fixation and external splinting until satisfactory healing of the osteotomy; another option is to curette and bone-graft or cement the lesion.

In summary, the contraindications of using this technique are marked osteolytic tumors and extensive involvement of the articular surface or absence of tumor free subchondral bone and in revision surgery, after a previous freezing or other biological recycling.

This study has some limitations; it is a retrospective study and it was conducted in small number of patients. We are hopeful that we can report the outcome of a larger cohort of children in the future.

## 5. Conclusion

Epiphyseal preservation and reconstruction by frozen bone autograft after malignant bone tumor resection are a good choice for treatment of a child with primary/secondary bone tumor, with good response to chemotherapy, with nonosteolytic lesion, without involvement of the articular cartilage or the subchondral bone. This method is easy, effective, biological, and comparatively inexpensive. It allows immediate mobilization of joints and possible cryoimmune effects and affords long term functional outcome. Pedicle freezing and hemicortical resection in addition to marginal resection are attempts providing a more biological reconstruction, thus enhancing a more rapid and satisfactory functional recovery after tumor resection in children.

## Figures and Tables

**Table 1:** Descriptive criteria of all cases.

Number	Age	Sex	Diagnosis	Location	Outcome	Freezing	Margin	Histological response	Function	FU
1	11	M	Osteosarcoma	Femur	CDF	Free freezing	Marginal	RH III/IV	Excellent	70
2	10	M	Osteosarcoma	Femur	CDF	Free freezing	Marginal	RH III/IV	Excellent	66
3	16	M	Osteosarcoma	Femur	CDF	Free freezing^*∗*^	Marginal	RH IV/IV	Excellent	63
4	6	F	Osteosarcoma	Femur	CDF	Free freezing	marginal	RH III/IV	Excellent	54
5	13	F	Osteosarcoma	Femur	CDF	Free freezing^*∗*^	Marginal	RH III/IV	Excellent	53
6	16	M	Undifferentiated round cell sarcoma	Calcaneus	CDF	Pedicle freezing	Wide	Total necrosis RH IV/IV	Excellent	53
7	11	F	Osteosarcoma	Tibia	CDF	Free freezing^*∗*^	Marginal	RH III/IV	Excellent	95
8	12	F	Osteosarcoma	Tibia	CDF	Pedicle freezing	Marginal	Total necrosis RH IV/IV	Excellent	87
9	16	F	Osteosarcoma	Tibia	NED	Free freezing	Wide	RH III/IV	Excellent	155
10	8	F	Osteosarcoma	Femur	NED	Free freezing	Wide	RH IV/IV	Excellent	90
11	13	F	osteosarcoma	femur	NED	pedicle freezing	Wide	RH III/IV	excellent	79
12	14	M	osteosarcoma	tibia	NED	pedicle freezing	Marginal	a few viable tumor cells	excellent	85
13	15	M	osteosarcoma	femur	NED	free freezing	Wide	RH III/IV	excellent	69
14	15	M	osteosarcoma	tibia	NED	pedicle freezing	Wide	RH III/IV	excellent	54
15	10	F	Osteosarcoma	tibia	NED	pedicle freezing	Marginal	RH III/IV	excellent	50
16	6	M	osteosarcoma	femur	AWD	free freezing	Wide	RH III/IV	poor	58
17	18	F	osteosarcoma	tibia	AWD	pedicle freezing	Wide	RH III/IV	excellent	98
18	11	M	Ewing's sarcoma	tibia	DOD	free freezing	Wide	RH III/IV	excellent	32

*The following abbreviations were used*; CDF: continuous disease free. NED: no evidence of disease. AWD: alive with the disease. DOD: died of the disease. FU: follow up. (*∗*): refers to cases who had a hemicortical resection.

RH: Rosen and Huvos Grade [15].

Grade I, little or no effect of chemotherapy noted; Grade II, a partial response to chemotherapy with greater than 50% tumor necrosis noted and attributable to preoperative chemotherapy; however, some histologic sections demonstrated areas of viable tumor; Grade III, greater then 90% tumor necrosis attributable to preoperative chemotherapy; however, foci of what appear to be viable tumor are seen in some histologic sections; and Grade IV, no viable-appearing tumor cells noted in any of the histologic sections.

**Table 2:** Summary of different limb reconstruction methods used in children.

Item of classification	Our Study	Reconstruction by expandable prosthesis [20]	Resurfaced allograft- prosthetic composite [21]	Epiphyseal preservation and allograft reconstruction [10]	Extracorporeal irradiation [1]	Stanmore noninvasive extendible endoprosthesis [3]
Number of patients	18	38	19	35	101	55

Mean follow-up (months)	72.8	113	78	108	52.8 (median)	41.2

Recurrence	5.5%		0%	9%	4.9%	0%

Infection	11.1%	47%	Deep 5% Superficial 0%	5.7%	5.9%	10.9%

Fracture	11.1%	15.7%	32%	31.4%	—	5.4%

Amputation	5.5%	8.5%	5.2%		3.9%	1.8%

Other complications	Nonunion 11.1%	Aseptic loosening 28% Dislocation 16.6%	Nonunion 10.5% Failure of extensor mechanism 14.5%	Nonunion 8.5%	Distant recurrence 19.8%	Persistent foot drop 3.6% Gearbox failure 5.4%

Overall survival	94.4%	55%	84%	86%	(80.8%~85.7%)	81.8%

Mean LLD	22 mm	37 mm	19 mm			

Overall complication rate	33.3%	58%		54%	35.4%	29.1%

Functional outcome	Excellent in 94.4% Poor in 5.5%	Excellent and good in 71% Poor in 29%	In 13 patients, excellent and good in 62% and fair in 38%		Will be reported later	Mean MSTS score 24.7
